# Effect of Test and Treat on clinical outcomes in Nigeria: A national retrospective study

**DOI:** 10.1371/journal.pone.0284847

**Published:** 2023-08-22

**Authors:** Marie-Claude C. Lavoie, Akipu Ehoche, Natalia Blanco, Ibrahim Ahmed El-Imam, Ademola Oladipo, Ibrahim Dalhatu, Solomon Odafe, Sylvia Adebajo, Alexia H. Ng, Laura Rapoport, Jonathan G. Lawton, Christopher Obanubi, Denis Onotu, Sadhna Patel, Akudo Ikpeazu, Greg Ashefor, Bashorun Adebobola, Mary Adetinuke Boyd, Gambo Aliyu, Kristen A. Stafford

**Affiliations:** 1 Division of Global Health Sciences, Department of Epidemiology and Public Health, University of Maryland School of Medicine, Baltimore, MD, United States of America; 2 Institute of Human Virology, University of Maryland School of Medicine, Baltimore, Maryland, United States of America; 3 Center for International Health Education and Biosecurity, University of Maryland School of Medicine, Baltimore, Maryland, United States of America; 4 Center for International Health Education and Biosecurity, MGIC-an Affiliate of the University of Maryland Baltimore, Abuja, Nigeria; 5 Centers for Disease Control and Prevention, CGH/DGHT, Abuja, Nigeria; 6 National AIDS and STI Control Programme-Federal Ministry of Health, Abuja, Federal Capital Territory, Nigeria; 7 National Agency for the Control of AIDS, Abuja, Nigeria; Nigerian Institute of Medical Research, NIGERIA

## Abstract

**Background:**

In Nigeria, results from the pilot of the Test and Treat strategy showed higher loss to follow up (LTFU) among people living with HIV compared to before its implementation. The aim of this evaluation was to assess the effects of antiretroviral therapy (ART) initiation within 14 days on LTFU at 12 months and viral suppression.

**Methods:**

We conducted a retrospective cohort study using routinely collected de-identified patient-level data hosted on the Nigeria National Data Repository from 1,007 facilities. The study population included people living with HIV age ≥15. We used multivariable Cox proportional frailty hazard models to assess time to LTFU comparing ART initiation strategy and multivariable log-binomial regression for viral suppression.

**Results:**

Overall, 26,937 (38.13%) were LTFU at 12 months. Among individuals initiated within 14 days, 38.4% were LTFU by 12 months compared to 35.4% for individuals initiated >14 days (p<0.001). In the adjusted analysis, individuals who were initiated ≤14 days after HIV diagnosis had a higher hazard of being LTFU (aHR 1.15, 95% CI 1.10–1.20) than individuals initiated after 14 days of HIV diagnosis. Among individuals with viral load results, 86.2% were virally suppressed. The adjusted risk ratio for viral suppression among individuals who were initiated ≤14 days compared to >14 days was not statistically significant.

**Conclusion:**

LTFU was higher among individuals who were initiated within 14 days compared to greater than 14 days after HIV diagnosis. There was no difference for viral suppression. The provision of early tailored interventions to support newly diagnosed people living may contribute to reducing LTFU.

## Introduction

Approximately 38 million people are living with HIV globally, 12.6 million of whom are awaiting treatment [1]. To tackle this widespread public health challenge, the Joint United Nations Programme on HIV/AIDS (UNAIDS) established the 95-95-95 global targets calling for 95% of all people who are living with HIV to know their status, 95% of people who know their status to be on treatment, and 95% of people on treatment to achieve viral suppression by 2025 [2]. At the end of 2020, 84% of people living with HIV globally knew their status, 87% of them received antiretroviral therapy (ART), and 90% of those were virally suppressed, leaving critical areas where progress is needed at a global level [3].

In Nigeria, HIV prevalence among adults aged 15–64 years is estimated at 1.4%, according to the latest HIV population-based survey [4]. With over 1.9 million people living with HIV, Nigeria has the fourth-largest population of people living with HIV [4]. In Nigeria, the UNAIDS HIV 95-95-95 target achievements were estimated to be 46.9%, 96.4%, and 80.9% at the end of 2018 [4]. The large gap in the first 95 led to large scale Surge efforts in Nigeria to find people living with HIV who did not know their status and link them to care [5]. By 2020, the country had moved closer to achieving the UNAIDS HIV 95-95-95 goal with an estimated 90% of all people living with HIV identified and 90% of all people living with initiated on ART [6].

In 2015, the World Health Organization (WHO) recommended initiating ART among all adults living with HIV irrespective of the WHO clinical stage or CD4 cell count [7], an initiative known as “Treat All”. This guideline was developed responding to the mounting scientific evidence showing that earlier ART initiation reduced mortality, morbidity, and HIV transmission [8–10]. Following these guidelines, concerns related to pre-ART loss to follow-up were growing and the “Test and Treat” strategy was developed; recommending rapid or same day ART initiation following a positive HIV test result [11]. Some studies have shown that the Test and Treat approach is associated with high retention in care and viral suppression [12–14]. The highest reported levels of viral load suppression in sub-Saharan Africa were under Test and Treat strategies combined with strong linkage to care programs [15]. In contrast, other studies found no effect or higher loss to follow up under Test and Treat [16, 17].

In 2015–2016, the Nigeria Federal Ministry of Health (FMOH) piloted the Test and Treat policy in selected government areas [17]. FMOH in collaboration with the University of Maryland Baltimore (UMB) and the Centers for Disease Control and Prevention (CDC) conducted the evaluation of this phase pilot of Test and Treat in Nigeria [17]. The results showed that 34% of patients who initiated ART under the Test and Treat strategy were lost to follow-up (LTFU) at 12 months post-ART initiation compared to 19% among individuals initiated on ART before Test and Treat [17]. In 2016, the FMOH guidelines recommended that all individuals diagnosed with HIV be offered ART and initiated promptly on treatment (preferably within two weeks). The effects of Test and Treat at the national level on retention and viral suppression at a later implementation stage of the guidelines remained unknown. To fill this gap, we assessed the effects of rapid ART initiation (within 14 days) on LTFU at 12 months and viral suppression across 1,007 facilities in Nigeria.

## Methods

### Study design and setting

We conducted a retrospective cohort study using routinely collected de-identified patient-level data hosted on the Nigeria National Data Repository (NDR) from all 1,007 PEPFAR-CDC supported facilities located across 15 states. HIV prevalence in the included states varied from 0.2% to 4.8% [4].

### Study population

The study population included people living with HIV age ≥15 years receiving HIV services in health facilities supported by PEPFAR-CDC programs. Patients were eligible if they were initiated on ART between April 2018 and March 2019. The study period includes up to March 2020 to enable a minimum of 12 months of follow-up. Pregnant women were excluded as their HIV care during pregnancy is provided at antenatal clinics which do not uniformly upload data to the NDR, and inclusion could overestimate LTFU and underestimate viral suppression.

### Variables

The exposure of interest was ART initiation within 14 days following HIV diagnosis. The primary outcome was time to LTFU within 12 months of ART initiation, defined as no drug pick-up for greater than 28 days after a missed drug pick-up appointment and the patient was not dead, transferred out, or did not return by the end of the study period (e.g., intermittent interruption in treatment was not classified as a loss to follow-up). Our secondary outcome was viral suppression, defined as HIV-RNA <1,000 copies/ml for the most recent viral load result between 4 and 12 months after HIV diagnosis. Additional analyses using <200 copies/ml cut point for viral suppression were also performed. Covariates included demographic characteristics (age, sex), clinical variables (ART regimen at the time of entry into the cohort), and facility volume (number of individuals who were newly initiated on ART at the facility during the evaluation period). Dolutegravir (DTG) as first-line option for treatment was introduced in 2016–2017 in Nigeria [18, 19].

### Data collection

Demographic, clinical, and laboratory data were extracted from the NDR which is an integrated repository for HIV data from multiple Electronic Medical Records (EMR) systems used for HIV care and treatment programs in Nigeria. The Nigeria FMOH coordinates and oversees the management of the NDR. All HIV service delivery partners provide technical assistance to healthcare facilities for EMR implementation and data exchange between the EMR systems and the NDR. The NDR has built-in automated data quality checks prompting implementing partners for further investigation and data quality improvement when needed.

### Statistical analysis

Categorical covariates were compared by ART initiation strategy (≤14 days versus >14 days following HIV diagnosis), ART retention (retained versus LTFU), and viral suppression (suppressed versus unsuppressed) using Chi-square tests. Time to LTFU was examined using Kaplan-Maier methods by stratum of ART initiation strategy (≤14 days versus >14 days following HIV diagnosis) and compared using a log-rank test. We used multivariable Cox proportional frailty hazard models adjusting for clustering within a facility to assess time to LTFU comparing ART initiation strategy. Individuals not LTFU were censored at 12-months. Individuals who were transferred out or died were excluded from the analysis. Viral suppression comparing ART initiation strategies was estimated using multivariable log-binomial regression accounting for clustering within a facility. In the multivariable model, variables significant (p<0.05) in the bivariate analysis or significant in the scientific literature were included. Statistical analyses were conducted using SAS version 9.4 (SAS Institute Inc., Cary, NC) and R.

### Ethical consideration

The evaluation study protocol was approved by the National Health Research Ethics Committee Nigeria (NHREC) and the Institutional Review Board of the University of Maryland, Baltimore. The regulatory authorities determined this study as non-human subject’s research. The requirements for written informed consent were waived as this evaluation was conducted on de-identified data and involved no more than minimal risk. This protocol was reviewed in accordance with CDC’s human research protection procedures. Investigators did not interact with human subjects or access unique personally identifiable data.

## Results

### Characteristics of the evaluation population

**Fig 1:**
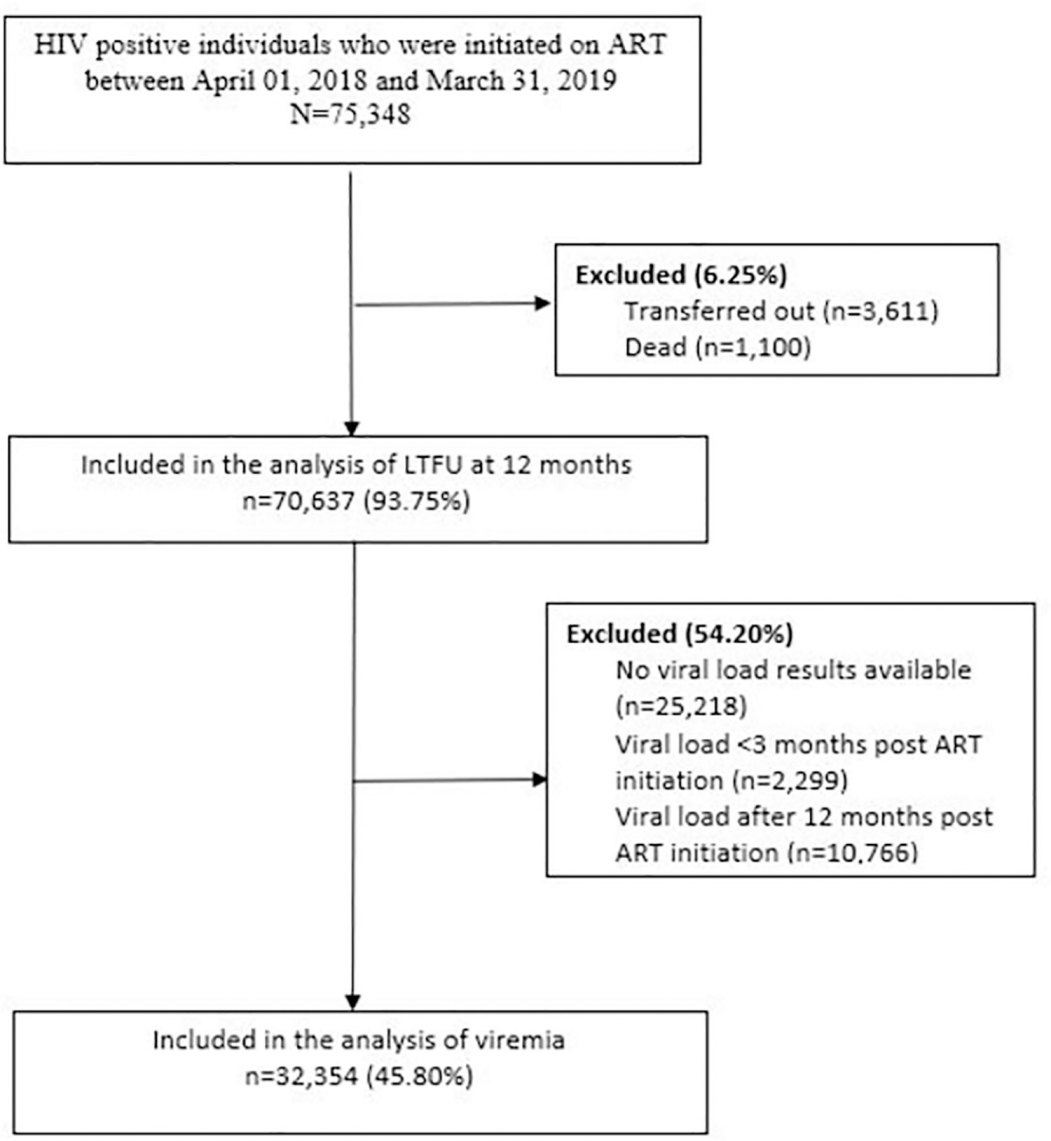
Flow diagram of individuals included in the analysis.

A total of 75,348 patients were included in the analysis, of whom 67,855 (90.1%) were initiated within 14 days (Table 1). Most individuals (53.8%) were aged between 25 and 39 years. The cohort was predominantly female (69.6%), and 85.3% were initiated on non-nucleoside reverse transcriptase inhibitor (NNRTI)–based regimens. Over ninety-two percent of the study sample received HIV services in a facility that initiated fewer than 500 people on ART during the evaluation period (facility volume). The distribution between individuals who were initiated within 14 days following HIV diagnosis and >14 days differed on age, ART regimen, facility volume, and state (all p<0.001). Sex was not associated with ART initiation strategy (Table 1). For individuals initiated after 14 days, the median time to ART initiation was 131 days. From the initial evaluation population (N = 75,348), a total of 4,711 (6.25%) were excluded as 3,611 (4.79%) were transferred out to another facility, and 1,100 (1.46%) had died after ART initiation (Fig 1).

**Table 1:** Baseline characteristics of HIV patients initiated on antiretroviral therapy either ≤14 days or > 14 days following HIV diagnosis between April 1, 2018 and March 31, 2019.

	ART Initiation ≤ 14 days Following HIV diagnosis (n = 67855) n (%)	ART Initiation > 14 days following HIV diagnosis (n = 7493) n (%)	Total	P-value
			(N = 75348) n (%)	
**Age Category**				
15–19	1294(1.91)	144(1.92)	1438(1.91)	<0.001
20–24	6165(9.09)	486(6.49)	6651(8.83)	
25–29	11461(16.89)	929(12.4)	12390(16.44)	
30–34	13421(19.78)	1436(19.16)	14857(19.72)	
35–39	11975(17.65)	1356(18.1)	13331(17.69)	
40–44	9107(13.42)	1231(16.43)	10338(13.72)	
45–49	5686(8.38)	709(9.46)	6395(8.49)	
50+	8746(12.89)	1202(16.04)	9948(13.2)	
**Sex**				
Female	47210(69.57)	5201(69.41)	52411(69.56)	0.78
Male	20645(30.43)	2292(30.59)	22937(30.44)	
**ART regimen at time of entry to cohort**				
DTG	9227(13.6)	755(10.08)	9982(13.25)	<0.001
NNRTI (EFV or NVP)	57664(84.98)	6592(87.98)	64256(85.28)	
PI	374(0.55)	106(1.41)	480(0.64)	
Other	590(0.87)	40(0.53)	630(0.84)	
**Facility Volume**				
0–499	62793(92.54)	6714(89.6)	69507(92.25)	<0.001
500–999	5062(7.46)	779(10.4)	5841(7.75)	
**State**				
Benue	17227(25.39)	1891(25.24)	19118(25.37)	<0.001
Delta	2737(4.03)	411(5.49)	3148(4.18)	
Ekiti	550(0.81)	63(0.84)	613(0.81)	
Enugu	2756(4.06)	285(3.8)	3041(4.04)	
FCT	4823(7.11)	531(7.09)	5354(7.11)	
Gombe	1582(2.33)	98(1.31)	1680(2.23)	
Imo	2592(3.82)	79(1.05)	2671(3.54)	
Kaduna	4691(6.91)	542(7.23)	5233(6.95)	
Katsina	1349(1.99)	127(1.69)	1476(1.96)	
Kogi	1335(1.97)	119(1.59)	1454(1.93)	
Lagos	3898(5.74)	295(3.94)	4193(5.56)	
Nasarawa	4841(7.13)	348(4.64)	5189(6.89)	
Ogun	2314(3.41)	441(5.89)	2755(3.66)	
Ondo	1755(2.59)	284(3.79)	2039(2.71)	
Osun	756(1.11)	195(2.6)	951(1.26)	
Oyo	3181(4.69)	325(4.34)	3506(4.65)	
Plateau	3246(4.78)	700(9.34)	3946(5.24)	
Rivers	8222(12.12)	759(10.13)	8981(11.92)	

*Chi-square test

### Loss to follow-up

**Fig 2:**
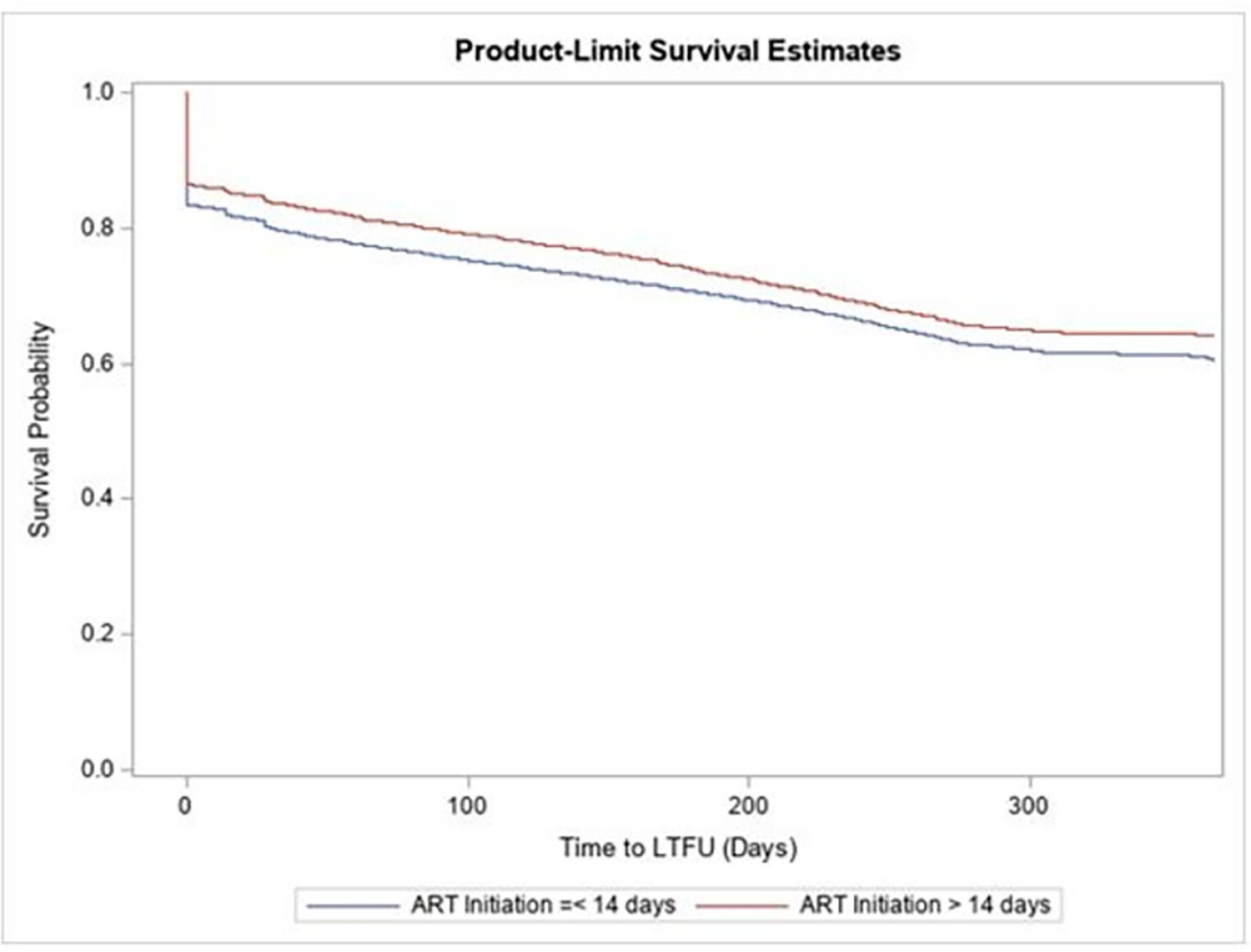
Kaplan-Meir survival curves showing time lost to follow-up by ART initiation (≤14 days vs >14 days). Log-rank test, p<0.001.

In the Kaplan-Meier analysis, patients initiated within 14 days had a greater probability of early LTFU than those initiated >14 days and had a higher overall probability of LTFU (p<0.001). Both curves were parallel and flattened around the last quarter of the 12 months (Fig 2). Among the remaining 70,637 patients, 26,937 (38.13%) were LTFU at 12 months. Among individuals initiated within 14 days, 38.4% were LTFU by 12 months compared to 35.4% for individuals initiated >14 days (p<0.001) (Table 2). Overall, the incidence rate of LTFU was 49 per 1,000 person-months; 50 per 1,000 person-months for individuals initiated within 14 days compared to 43 per 1,000 person-months for individuals initiated after 14 days. The individuals who were initiated within 14 days had 1.2 times the rate of being LTFU compared to individuals initiated after 14 days (IRR = 1.16, 95%CI 1.11–1.21).

**Table 2:** Baseline characteristics among HIV-positive patients initiated on antiretroviral therapy between April 1, 2018 and March 31, 2019 by loss to follow status at 12 months.

	Lost to follow up (n = 26937) n (%)	Not lost to follow up (n = 43700) n (%)	Total (N = 70637) n (%)	P-value*
**ART initiation strategy**				
ART Initiation ≤ 14 days	24390(38.44)	39052(61.56)	63442(89.81)	<0.001
ART Initiation > 14 days	2547(35.4)	4648(64.6)	7195(10.19)	
**Age Category**				
15–19	498(36.92)	851(63.08)	1349(1.91)	<0.001
20–24	2587(41.81)	3601(58.19)	6188(8.76)	
25–29	4703(40.67)	6862(59.33)	11565(16.37)	
30–34	5283(37.98)	8627(62.02)	13910(19.69)	
35–39	4796(38.37)	7704(61.63)	12500(17.70)	
40–44	3570(36.77)	6138(63.23)	9708(13.74)	
45–49	2120(35.03)	3932(64.97)	6052(8.57)	
50+	3380(36.09)	5985(63.91)	9365(13.26)	
**Sex**				
Female	18774(38.13)	30466(61.87)	49240(69.71)	0.96
Male	8163(38.15)	13234(61.85)	21397(30.29)	
**ART regimen at time of entry to cohort**				
DTG	3318(35.42)	6049(64.58)	9367(13.26)	<0.001
NNRTI (EFV or NVP)	23275(38.62)	36984(61.38)	60259(85.31)	
PI	179(43.13)	236(56.87)	415(0.59)	
Other	165(27.68)	431(72.32)	596(0.84)	
**Facility Volume**				
0–499	25000(38.43)	40051(61.57)	65051(92.09)	<0.001
500–999	1937(34.68)	3649(65.32)	5586(7.91)	
**State**				
Benue	5301(29.18)	12868(70.82)	18169(25.72)	<0.001
Delta	1302(42.01)	1797(57.99)	3099(4.39)	
Ekiti	205(36.61)	355(63.39)	560(0.79)	
Enugu	871(32.89)	1777(67.11)	2648(3.75)	
FCT	2149(44.44)	2687(55.56)	4836(6.85)	
Gombe	625(37.47)	1043(62.53)	1668(2.36)	
Imo	907(35.94)	1617(64.06)	2524(3.57)	
Kaduna	1561(33.42)	3110(66.58)	4671(6.61)	
Katsina	392(33.36)	783(66.64)	1175(1.66)	
Kogi	269(19.17)	1134(80.83)	1403(1.99)	
Lagos	1761(43.69)	2270(56.31)	4031(5.71)	
Nasarawa	2099(43.79)	2694(56.21)	4793(6.79)	
Ogun	1206(44.44)	1508(55.56)	2714(3.84)	
Ondo	588(34.21)	1131(65.79)	1719(2.43)	
Osun	462(49.89)	464(50.11)	926(1.31)	
Oyo	1814(53.35)	1586(46.65)	3400(4.81)	
Plateau	1427(36.6)	2472(63.4)	3899(5.52)	
Rivers	3998(47.58)	4404(52.42)	8402(11.89)	

*Chi-square test

### Factors associated with loss to follow-up

In the bivariate analysis, LTFU differed significantly by ART initiation strategy (≤14 days vs >14 days), age, ART regimen, and facility volume. In the Cox proportional hazard crude and adjusted analysis, individuals who were initiated ≤14 days after HIV diagnosis had a higher hazard of being LTFU (uHR 1.12, 95% CI 1.08 to 1.17, aHR 1.15, 95% CI 1.10–1.20) compared to individuals initiated after 14 days of HIV diagnosis after adjusting for clustering within the facility, sex, age, facility volume, and ART regimen. There was a higher hazard of LTFU among younger individuals (age groups 20–24, 25–29, 30–34, and 35–39) compared to older adults (50+) (aHR 1.29 95% CI 1.23–1.36, 1.21 95% CI 1.15–1.26, 1.11 95% CI 1.06–1.16, and 1.09 95% CI 1.04–1.14 respectively), and men compared to women (aHR 1.08, 95% CI 1.05–1.11). Individuals on dolutegravir (DTG) had a lower hazard of being LTFU than those on an NNRTI containing regimen (aHR 0.80, 95% CI 0.77–0.83) (Table 3).

**Table 3:** Unadjusted and adjusted hazard ratios (uHR/aHR) with 95% confidence intervals (95% CI) from Cox proportional hazards model assessing the association between baseline characteristics and rate of loss to follow-up among test and start patients.

	uHR	95% CI	P-value	aHR*	95% CI	P-value
**ART initiation strategy**						
≤ 14 days	1.12	1.08–1.17	0.00	1.15	1.10–1.20	<0.001
> 14 days	Ref			Ref		
**Age categories**						
15–19	1.01	0.91–1.104	0.93	1.10	1.00–1.21	0.06
20–24	1.18	1.12–1.245	0.00	1.29	1.23–1.36	<0.001
25–29	1.15	1.10–1.20	0.00	1.21	1.15–1.26	<0.001
30–34	1.06	1.01–1.10	0.01	1.11	1.06–1.16	<0.001
35–39	1.07	1.03–1.12	0.00	1.09	1.04–1.14	<0.001
40–44	1.02	0.97–1.07	0.47	1.05	1.00–1.10	0.05
45–49	0.96	0.91–1.02	0.16	0.97	0.92–1.03	0.29
50+	Ref			Ref		
**Sex**						
Female	Ref			Ref		
Male	1.00	0.98–1.03	0.89	1.08	1.05–1.11	<0.001
**Facility Volume**						
0–499	Ref			Ref		
500–999	0.86	0.86–0.82	0.00	0.85	0.45–1.60	0.61
**ART regimen at time of entry to cohort**						
NNRTI (EFV or NVP)	Ref			Ref		
DTG	0.92	0.89–0.96	0.00	0.80	0.77–0.83	<0.001
PI	1.19	1.03–1.38	0.02	1.49	1.28–1.74	<0.001
Other	0.67	0.57–0.78	0.00	1.03	0.87–1.22	0.72

* Adjusted model clustered by facility (frailty model)

### Factors associated with viral suppression

Among the 70,637 individuals, 32,354 (45.8%) had a viral load available between 4- and 12-months following HIV diagnosis. Among them, 27,904 (86.2%) were virally suppressed, defined as HIV-RNA <1000 copies/ml for the most recent viral load result (Table 4). The crude and adjusted risk ratios for viral suppression among individuals who were initiated ≤14 days compared to >14 days were consistently not statistically significant (uRR 1.01, 95% CI 1.00–1.03, aRR 1.01, 95% CI 1.00–1.03) (Table 5). S1 and S2 Tables show similar results when assessing viral suppression as HIV-RNA <200 copies/ml.

**Table 4:** Factors associated with viral load suppression (<1000 copies/ml) among HIV-positive patients initiated on antiretroviral therapy between April 1, 2018 and March 31, 2019.

	Not Suppressed (n = 4450) n (%)	Suppressed (n = 27904) n (%)	Total (N = 32354) n (%)	P-value*
**ART initiation strategy**				
ART Initiation ≤ 14 days	3964(13.70)	24961(86.30)	28925(89.40)	0.98
ART Initiation > 14 days	486(14.17)	2943(85.83)	3429(10.60)	
**Age Category**				
15–19	155(24.88)	468(75.12)	623(1.93)	<0.001
20–24	385(14.81)	2215(85.19)	2600(8.04)	
25–29	685(13.69)	4319(86.31)	5004(15.47)	
30–34	870(14.08)	5307(85.92)	6177(19.09)	
35–39	806(14.36)	4806(85.64)	5612(17.35)	
40–44	578(12.61)	4007(87.39)	4585(14.17)	
45–49	393(12.85)	2665(87.15)	3058(9.45)	
50+	578(12.31)	4117(87.69)	4695(14.51)	
**Sex**				
Female	3123(13.83)	19459(86.17)	22582(69.80)	0.95
Male	1327(13.58)	8445(86.42)	9772(30.20)	
**ART regimen at time of entry to cohort**				
DTG	611(11.68)	4619(88.32)	5230(16.16)	<0.001
NNRTI (EFV or NVP)	3742(14.06)	22864(85.94)	26606(82.23)	
PI	36(20.22)	142(79.78)	178(0.55)	
Other	61(17.94)	279(82.06)	340(1.05)	
**Facility Volume**				
0–499	4032(13.71)	25375(86.29)	29407(90.89)	0.09
500–999	418(14.18)	2529(85.82)	2947(9.11)	
**State**				
Benue	930(9.64)	8720(90.36)	9650(29.83)	<0.001
Delta	167(13.2)	1098(86.8)	1265(3.91)	
Ekiti	47(14.07)	287(85.93)	334(1.03)	
Enugu	144(13.51)	922(86.49)	1066(3.29)	
FCT	405(16.34)	2073(83.66)	2478(7.66)	
Gombe	57(14.43)	338(85.57)	395(1.22)	
Imo	224(18.48)	988(81.52)	1212(3.75)	
Kaduna	165(20.37)	645(79.63)	810(2.50)	
Katsina	101(16.83)	499(83.17)	600(1.85)	
Kogi	158(27.92)	408(72.08)	566(1.75)	
Lagos	232(11.89)	1720(88.11)	1952(6.03)	
Nasarawa	379(16.76)	1883(83.24)	2262(6.99)	
Ogun	168(11.25)	1325(88.75)	1493(4.61)	
Ondo	133(13.39)	860(86.61)	993(3.07)	
Osun	57(11.52)	438(88.48)	495(1.53)	
Oyo	218(14.69)	1266(85.31)	1484(4.59)	
Plateau	233(13.17)	1536(86.83)	1769(5.47)	
Rivers	632(17.9)	2898(82.1)	3530(10.91)	

*Chi-square test

**Table 5:** Unadjusted and adjusted risk ratios (uRR/aRR) with 95% confidence intervals (95% CI) from multivariable log poisson regression assessing the hazard of viral load suppression (<1000 copies/ml) after initiating antiretroviral therapy based on timing of ART initiation.

	uRR	95% CI	P-value	aRR	95% CI	P-value
**ART initiation strategy**						
ART Initiation ≤ 14 days	1.01	1.0–1.03	0.11	1.01	1.0–1.03	0.13
ART Initiation > 14 days	Ref		.	Ref		
**Age categories**						
15–19	0.85	0.81–0.89	0.00	0.86	0.82–0.89	<0.001
20–24	0.96	0.95–0.98	0.00	0.97	0.95–0.99	<0.001
25–29	0.98	0.97–1.00	0.02	0.98	0.97–1.00	0.04
30–34	0.98	0.96–0.99	0.00	0.98	0.97–0.99	0.01
35–39	0.98	0.96–0.99	0.00	0.98	0.96–1.00	0.01
40–44	1.00	0.98–1.01	0.67	1.00	0.98–1.01	0.84
45–49	0.99	0.98–1.01	0.54	1.00	0.98–1.01	0.58
50+	Ref			Ref		
**Sex**						
Female	Ref			Ref		
Male	1.00	0.99–1.01	0.44	1.00	0.98–1.01	0.37
**Facility Volume**						
0–499	Ref			Ref		
500–999	1.01	0.95–1.06	0.81	1.01	0.95–1.06	0.82
**ART regimen at time of entry to cohort**						
NNRTI (EFV or NVP)	Ref			Ref		
DTG	1.04	1.02–1.05	0.00	1.03	1.02–1.05	<0.001
PI	0.94	0.88–1.00	0.04	0.95	0.89–1.01	0.09
Other	0.99	0.94–1.06	0.86	0.99	0.93–1.05	0.74

## Discussion

Despite early findings from the pilot phase of the Test and Treat guidelines in Nigeria [17], subsequent robust evaluations are scarce. This evaluation adds novel data on the effects of Test and Treat on LTFU and viral suppression at a later implementation stage. Overall, 90% were initiated within 14 days, and 35% were LTFU at 12 months following ART initiation irrespective of the time of ART initiation following HIV diagnosis. Patients who were initiated on ART within 14 days had a higher hazard of being LTFU than those initiated after 14 days following HIV diagnosis.

Similar to the 2015–2016 pilot evaluation, our evaluation found a high percentage of LTFU by 12 months. In contrast, other earlier studies in Nigeria have reported a lower LTFU [20, 21]. Most of the attrition occurs shortly after treatment initiation, regardless of the timing of ART initiation, and then follows a parallel time-to-event pattern over time. This finding is similar to those previously reported by Williams Sherlock et al. (2022) that found a significantly higher proportion of individuals experiencing treatment interruption among those who were newly initiated on ART (<3 months) compared to those on ART for more than three months across 45 countries supported by PEPFAR [22]. In terms of potential reasons for this high LTFU irrespective of ART strategy, it is plausible that a large proportion of individuals identified as LTFU may be seeking HIV services at different facilities (silent transfer), or they may still be active at the original facility but misclassified as LTFU due to administrative errors or oversights in documenting clinic visits as previously reported in SSA [23, 24]. In Nigeria, according to the latest population-based survey, 95% of the HIV-positive adults who reported initiating ART within the last 12 months or more preceding the survey were on ART [4]. Based on this data, we suspect that a proportion of individuals identified as LTFU may be loss not from clinical care but “loss to observation” [25] as they are receiving HIV care at a different facility or same facility (without proper clinical documentation). Efforts have been underway in Nigeria to increase access to ART and also retention [26], and additional interventions are being implemented to optimize longitudinal care and to reflect the accurate status of the client such as the use of biometrics. The effects of these interventions on continuous engagement in care are yet to be determined.

Similar to the pilot evaluation [17], individuals who were initiated within 14 days were significantly more likely to be LTFU than those initiated after 14 days. Studies on the effects of the Test and Treat strategy on clinical outcomes have shown mixed results. In Malawi, researchers have found a positive effect on retention [14]. In contrast, researchers in South Africa found lower retention rates among patients initiating treatment under Test and Treat [16]. In Eswatini, researchers found that same day initiation was associated with unfavorable outcomes (death, LTFU, and viral failure) [27]. In a multi-country study conducted in SSA, same-day ART initiation was associated with significantly higher LTFU than delayed ART initiation.

For viral suppression, similar to previous studies, we found no difference in viral suppression between the patients initiated within 14 days (Test and Treat) and those initiated >14 days after HIV diagnosis. Viral load monitoring and viral suppression was higher in this evaluation compared to the pilot evaluation (43.0% vs 11% and 86.2% vs 79.0% respectively). Despite an improvement in viral load monitoring, implementation gaps persist in adhering to the national guideline that recommends that routine viral load monitoring should be conducted at 6- and 12-months following ART initiation [19]. Viral load measurement is a critical strategy to clinically manage people living with HIV on treatment and also at the population level to monitor the progress to reach the third 95 of UNAIDS in which 95% of individuals on ART are virally suppressed [2]. In recent years, Nigeria has made considerable investments in improving clinical documentation and viral load monitoring, including data quality assurance and improvement programs, expansion of PCR laboratories, training on the clinical importance of viral loads, and the creation of the NDR. The NDR hosted and centralized EMR data previously accessible only by physical chart review at facilities; this repository enabled feedback on data completeness and quality and enhanced monitoring of viral load cascade. Future evaluations are warranted to assess the effects of these investments have contributed to increasing viral load monitoring and documentation.

Consistent with other studies we found that being a male was associated with LTFU [28, 29]. DTG-based regimen was a protective factor against LTFU which may be explained by its high tolerability compared to other ART regimens and low rates of adverse events [30, 31]. The sample for this evaluation was drawn from the early phases of DTG rollout out in Nigeria when a small proportion of individuals were on DTG (13%). In 2020, approximately 80% are estimated to be on DTG-based regimen [32]. Based on our findings, it is likely that the scale-up of DTG has positively impacted HIV continuity of care.

The findings of this evaluation underscore the need to better understand and improve patient engagement and service delivery factors that promote continuity in care among individuals newly initiated on treatment, especially considering the large proportion of LTFU irrespective timing of ART initiation. Identification and implementation of co-interventions to effectively support ART initiation and continuous engagement in care (especially during the early months) are needed. Investigation into facility-level outcomes may also reveal facility-specific factors influencing retention.

The strengths of the study include a large representative sample of facilities and patients, including adolescents and adults, across 1,007 facilities in Nigeria which was possible due to the national patient-level data repository. This study is also subject to limitations. First, this study includes the use of programmatic data which is subject to inconsistent reporting among data entry personnel and errors in documentation, data entry, and extraction. Data quality assurance measures were implemented across all facilities which included standardized data collection tools and processes, automated validation checks and rules in the EMRs and Nigeria NDR, and facility and state-level monthly data review and on-site data quality assurance. Second, information obtained from a retrospective review of records were limited by unmeasured factors, such as CD4 counts or WHO stage, and we did not have data on reasons for delay ART initiation which may have affected our outcomes. However, under Test and Treat guidelines, all individuals, irrespective of CD4 count and WHO stages, are recommended to initiate ART on the same day of HIV diagnosis or as early as possible. Third, this analysis included individuals who were initiated on ART therefore individuals who were diagnosed but not linked to treatment were not included in the analysis, which may have led to under-reporting of LTFU. However, this difference would be relatively small as more than 95% of newly identified people living with HIV were linked to treatment during this period in PEPFAR-supported sites [6]. On the other side, individuals who were identified as LTFU may have sought HIV services in other facilities, which may have led to an overestimation of LTFU. Fourth, for viral suppression, we were limited to a sub-set of the included population due to missing data on viral load results which may be partially explained by incomplete documentation in the EMR.

## Conclusions

This study provides evidence that viral load suppression is achievable regardless of timing of ART initiation and with continuity of care. While there was evidence of an association between rapid ART initiation following diagnosis and LTFU, a review of the guidelines is likely not warranted. Rather attention to counseling to improve knowledge of HIV, ART regimens, side effects and importance of adherence, as well as readiness assessment to commence lifelong treatment, and scale up of DTG-regimen appear critical components for retention and viral load suppression.

## Supporting information

S1 Table Factors associated with viral load suppression (HIV-RNA <200 copies/ml) among HIV-positive patients initiated on antiretroviral therapy between April 1, 2018 and March 31, 2019. (DOCX)

S2 Table Adjusted Risk Ratios (aRR) and 95% Confidence Intervals (95% CI) from multivariable log poisson regression modeling the probability of viral load suppression (HIV-RNA <200 copies/ml) after initiating antiretroviral therapy based on timing of ART initiation. (DOCX)
