# Supplementary material for: Effect of Test and Treat on clinical outcomes in Nigeria: A national retrospective study
Source: PLoS One. 2023 Aug 22;18(8):e0284847. doi: 10.1371/journal.pone.0284847 (PMC10443836; doi:10.1371/journal.pone.0284847)
Supplement: S1 Table — (DOCX) [file pone.0284847.s001.docx]

| **Table S1: Factors associated with viral load suppression (HIV-RNA <200 copies/ml) among HIV-positive patients initiated on antiretroviral therapy between April 1, 2018 and March 31, 2019** | | | | |
| --- | --- | --- | --- | --- |
|  |  |  |  |  |
|  | **Not Suppressed** | **Suppressed** | **Total** | **P-value** |
|  | **(N=6705)** | **(N=25649)** | **(N=32354)** |  |
| **Time to ART Start** |  |  |  |  |
| ART Initiation < 14 days | 5977(20.7) | 22948(79.3) | 28925 (89.4) | 0.452 |
| ART Initiation > 14 days | 728(21.2) | 2701(78.8%) | 3429 (10.6) |  |
| **Age Category** |  |  |  |  |
| 15-19 | 199(31.9) | 424(68.1%) | 623 (1.9) | <0.001 |
| 20-24 | 551(21.2) | 2049(78.8%) | 2600 (8.0) |  |
| 25-29 | 1052(21.0) | 3952(79%) | 5004 (15.5) |  |
| 30-34 | 1265(20.5) | 4912(79.5%) | 6177 (19.1) |  |
| 35-39 | 1217(21.7) | 4395(78.3%) | 5612 (17.3) |  |
| 40-44 | 882(19.2) | 3703(80.8%) | 4585 (14.2) |  |
| 45-49 | 601(19.7) | 2457(80.3%) | 3058 (9.5) |  |
| 50+ | 938(20.0) | 3757(80%) | 4695 (14.5) |  |
| **Sex** |  |  |  |  |
| Female | 4650(20.6) | 17932(79.4) | 22582 (69.8) | 0.38 |
| Male | 2055(21%.0) | 7717(79.0) | 9772 (30.2) |  |
| **ART regimen at time of entry to cohort** | |  |  |  |
| DTG | 988(18.9) | 4242(81.1) | 5230 (16.2) | <0.001 |
| NNRTI (EFV or NVP) | 5568(20.9) | 21038(79.1) | 26606 (82.2) |  |
| PI | 55(30.9) | 123(69.1) | 178 (0.6) |  |
| Other | 94(27.6) | 246(72.4) | 340 (1.1) |  |
| **Facility Volume** |  |  |  |  |
| 0 - 499 | 6077(20.7) | 23330(79.3) | 29407 (90.9) | 0.424 |
| 500 - 999 | 628(21.3) | 2319(78.7) | 2947 (9.1) |  |
| **State** |  |  |  |  |
| Benue | 1400(14.5) | 8250(85.5) | 9650 (29.8) | <0.001 |
| Delta | 259(20.5) | 1006(79.5) | 1265 (3.9) |  |
| Ekiti | 72(21.6) | 262(78.4) | 334 (1.0) |  |
| Enugu | 232(21.8) | 834(78.2) | 1066 (3.3) |  |
| FCT | 606(24.5) | 1872(75.5) | 2478 (7.7) |  |
| Gombe | 92(23.3) | 303(76.7) | 395 (1.2) |  |
| Imo | 340(28.1) | 872(71.9) | 1212 (3.7) |  |
| Kaduna | 220(27.2) | 590(72.8) | 810 (2.5) |  |
| Katsina | 163(27.2) | 437(72.8) | 600 (1.9) |  |
| Kogi | 194(34.3) | 372(65.7) | 566 (1.7) |  |
| Lagos | 346(17.7) | 1606(82.3) | 1952 (6.0) |  |
| Nasarawa | 567(25.1) | 1695(74.9) | 2262 (7.0) |  |
| Ogun | 278(18.6) | 1215(81.4) | 1493 (4.6) |  |
| Ondo | 219(22.1) | 774(77.9) | 993 (3.1) |  |
| Osun | 83(16.8) | 412(83.2) | 495 (1.5) |  |
| Oyo | 331(22.3) | 1153(77.7) | 1484 (4.6) |  |
| Plateau | 350(19.8) | 1419(80.2) | 1769 (5.5) |  |
| Rivers | 953(27.0) | 2577(73.0) | 3530 (10.9) |  |
