# Supplementary material for: Effect of Test and Treat on clinical outcomes in Nigeria: A national retrospective study
Source: PLoS One. 2023 Aug 22;18(8):e0284847. doi: 10.1371/journal.pone.0284847 (PMC10443836; doi:10.1371/journal.pone.0284847)
Supplement: S2 Table — (DOCX) [file pone.0284847.s002.docx]

| **Table S2: Adjusted Risk Ratios (aRR) and 95% Confidence Intervals (95% CI) from multivariable log Poisson regression modeling the probability of viral load suppression (HIV-RNA <200 copies/ml) after initiating antiretroviral therapy based on timing of ART initiation.** | | | | | | | | | |
| --- | --- | --- | --- | --- | --- | --- | --- | --- | --- |
|  | | **Unadjusted** | | |  | **Adjusted** | | |  |
|  |  | **RR** | **95% CI** | **P-value** |  | **RR** | **95% CI** | **P-value** |  |
| **Time to ART Start** | |  |  |  |  |  |  |  |  |
| ART Initiation < 14 days | | 1.02 | 1.0-1.04 | 0.08 |  | 1.02 | 1.0-1.04 | 0.09 |  |
| ART Initiation > 14 days | | Ref |  |  |  | Ref |  |  |  |
| **Age categories** | |  |  |  |  |  |  |  |  |
|  | 15-19 | 0.84 | 0.79-0.89 | <0.001 |  | 0.84 | 0.80-0.89 | <0.001 |  |
|  | 20-24 | 0.98 | 0.95-1.0 | 0.06 |  | 0.98 | 0.95-1.0 | 0.06 |  |
|  | 25-29 | 0.98 | 0.96-1.0 | 0.08 |  | 0.98 | 0.96-1.0 | 0.09 |  |
|  | 30-34 | 0.99 | 0.97-1.01 | 0.31 |  | 0.99 | 0.97-1.01 | 0.35 |  |
|  | 35-39 | 0.98 | 0.96-1.0 | 0.06 |  | 0.98 | 0.96-1.0 | 0.08 |  |
|  | 40-44 | 1.01 | 0.99-1.03 | 0.40 |  | 1.01 | 0.99-1.03 | 0.34 |  |
|  | 45-49 | 1.01 | 0.98-1.03 | 0.68 |  | 1.01 | 0.98-1.03 | 0.65 |  |
|  | 50+ | Ref |  |  |  | Ref |  |  |  |
| **Sex** | |  |  |  |  |  |  |  |  |
|  | Female | Ref |  |  |  | Ref |  |  |  |
|  | Male | 0.99 | 0.98-1.01 | 0.45 |  | 0.99 | 0.97-1.0 | 0.05 |  |
| **Facility Volume** | |  |  |  |  |  |  |  |  |
|  | 0 - 499 | Ref |  |  |  | Ref |  |  |  |
|  | 500 - 999 | 1.02 | 0.94-1.11 | 0.68 |  | 1.02 | 0.93-1.11 | 0.72 |  |
| **ART regimen at time of entry** | |  |  |  |  |  |  |  |  |
|  | NNRTI (EFV or NVP) | Ref |  |  |  | Ref |  |  |  |
|  | DTG | 1.03 | 1.02-1.05 | <0.001 |  | 1.03 | 1.01-1.05 | <0.001 |  |
|  | PI | 0.88 | 0.81-0.95 | <0.001 |  | 0.89 | 0.81-0.97 | 0.01 |  |
|  | Other | 0.99 | 0.92-1.06 | 0.71 |  | 0.98 | 0.92-1.05 | 0.65 |  |
